# Subclinical Rickets

**Published:** 2014

**Authors:** Tanveer Hussain Shah, Mukhtiar Hassan, Tahir Saeed Siddiqui

**Affiliations:** 1 Tanveer Hussain Shah, PhD, Department of Biochemistry & Health Science, Hazara University Mansehra, KPK, Pakistan.; 2 Mukhtiar Hassan, PhD, Department of Biochemistry & Health Science, Hazara University Mansehra, KPK, Pakistan.; 3 Tahir Saeed Siddiqui, FCPS, Ayub Medical College & Teaching Hospital, Abbottabad, Pakistan.

**Keywords:** Sub clinical, Camouflagic rickets, Hazarian school students, Physical indications, Daily meals, Synergistic effect

## Abstract

***Objective:*** The study on sub clinical rickets is unique in the sense that it has not been preplanned conducted anywhere especially in Pakistan. The objective of present study was to explore the prevalence, gender and geographical distribution of sub clinical rickets and their related factors among school students.

***Methods:*** Out of total participants, 189(90%) students were finally included in the study from rural, urban and suburban high schools of Hazara Division, KPK. The age of boys and girls students was 11 years to 16 years. Anthropometrics data along with daily intakes of meal and availability of sun shine was noted on record form. Sub clinical cases were diagnosed with abnormal biochemical findings without physical indications of rickets.

***Results:*** Sub clinical rickets was found in 51(27%) students, out of which 15(8%) were boys and 36(19%) girls. Geographically, 26 cases of sub clinical rickets were from rural schools, 16 of urban and 09 found in suburban school. All sub clinical cases had serum level of sunshine vitamin D in between ≥18nmol/l to ≤39 nmol/l, but none of them had parathyroid level increased from upper normal range. Estimated quantities of vitamin D, calcium and phosphorus in daily intakes meal of boy’s and girl’s student were almost same and found less than recommended amount.

***Conclusion:*** Sub clinical rickets is camouflagic rickets among Hazarian school students, especially in girl gender. The major cause contributed to this problem is lack of synergistic effect of Sunshine Vitamin D.

## INTRODUCTION

Poor mineralization of bone affects the growing skeleton and may cause rickets.  Chance of rickets is higher in adolescents due to more demand of nutrients.^2^ Vitamin D level in blood depends on its quantity received through foods and on endogenous synthesis in the body.^3^ In vitamin D deficiency cases, the serum calcium level falls due to impaired intestinal absorption and as a result mechanism of secondary hyperthyroidism takes place.^4^ Clinical vitamin D deficiency rickets is a widespread problem in children of Asian countries.^5^

Most of the studies were conducted about clinical rickets but no preplanned work has been done on sub clinical rickets anywhere, especially in Pakistan. Main objectives of this study were to explore the prevalence, gender and geographical distribution of sub clinical rickets and their related factors among school students.

## METHODS

This research study was approved by ethical committee of Ayub Medical College Abbottabad and Hazara University Mansehra. Before commencing this study, permission was obtained by higher authority of education and from parents of students. In this study 210 boys and girls student of age 11 years to 16 years who were apparently normal voluntarily participated from different government school of Hazara Division.

Students were divided into three groups, rural, urban and suburban on the basis of their school location and setting areas. After final screening, one hundred eighty nine students were included in the study who were having normal blood urea and creatinine level.

Demographic information such as age, weight and height, availability of sun shine and daily intake of vitamin D, calcium and phosphorus were recorded on data form. Clinical examination of students was done by expert doctor.

Blood sample was taken from each participant and serum was separated through centrifugation. Analysis of serum was performed to investigate the level of 25(OH) D, calcium, phosphorus, alkaline phosphatase and parathyroid hormone. Minitab statistical software was used for the analysis of data. Mean value/standard deviation was calculated and significant differences (<0.05) of parameters was evaluated among the groups.

## RESULTS

Out of 189 participants, 96 students were boys and 93 girls with almost same ratio. The mean age of boys was 13.83 ±1.58 and for girls 13.76±1.45 years with no statistical differences (>0.05). Number of included boys and girls students with their ages was same in all three geographical groups (>0.05) (Table-I). 

**Table-I:** Geographical and gender wise data of study participants.

***Groups***	***Boys***		***Girls***		***P value***
	***Numbers (%)***	***Age (years) Mean±SD***	***Numbers (%)***	***Age (years) Mean±SD***	
Rural	34(35.42)	13.69±1.69	33(35.48)	13.96±1.54	>0.05
Urban	30(31.25)	13.93±1.64	30(32.26)	13.75±1.42	>0.05
Suburban	32(33.33)	13.87±1.47	3032.26)	13.54±1.40	>0.05
Total	96	13.83±1.58	93	13.76±1.45	>0.05
P value	>0.05		>0.05		

Among 189 students, sub clinical rickets cases was detected in 51(27%), out of which 15(29%) boys and 36(71%) were girls. Geographically 26(51%) sub clinical cases from rural, 16(31%) urban and 09(18%) belonged to suburban schools. The significant differences (<0.05) was noted regarding geographical distribution and prevalence of sub clinical rickets between girls 36(19%) and boys 15(8%). Nutritional status shows that all participants of study (189) were taking less than required amount of vitamin D, calcium and phosphorus in their daily meal. Although estimated quantity of these nutrients taken by sub clinical cases in their meal was less than intake of normal cases, but no significant difference was seen (>0.05) (Table-II).

**Table-II:** Comparison of nutritional intakes by normal and sub clinical cases of different gender and groups

***Groups***	***Vitamin D(IU)*** ***Mean±SD***		***Calcium(mg)*** ***Mean±SD***		***Phosphorus (mg)*** ***Mean±SD***	
	***Boys***	***Girls***	***Boys***	***Girls***	***Boys***	***Girls***
Rural(Normal)	29.48±3.14	30.64±4.22	268.9±25.9	290.1±34.6	335.4±26.0	327.9±30.1
Rural(Sub clinical)	26.86±2.67	27.95±3.08	247.3±22.5	267.2±27.4	305.7±31.7	310.6±23.7
P value	0.050	0.055	0.053	0.051	0.052	0.088
Urban (Normal)	30.48±3.55	29.05±3.44	289.7±21.7	291.1±29.8	334.3±20.1	315.9±43.5
Urban (Sub clinical)	28.4±1.52	27.27± 1.19	251.8±30.7	268.5±28.3	296±29.50	297±14.6
P value	0.052	0.051	0.058	0.052	0.050	0.095
Suburban (Normal)	30.34±3.46	30.79±1.98	308.9±18.8	296±16.9	348.7±14.4	323.5±16.7
Suburban (Sub clinical)	27.67±1.53	28.33±2.42	241.7±29.3	265.8±28.4	297.3±23.7	300.8±21.1
P value	0.070	0.061	0.060	0.054	0.066	0.050

Significant difference (<0.05), Non significant difference (>0.05)

On the other hand no significant difference was found regarding daily intakes of vitamin D, calcium and phosphorus among rural, urban and suburban sub clinical groups of similar gender and in between different gender of similar area groups (>0.05).Vitamin D level noted in serum of rural girls was 23.42 nmol/l, urban 28.91 nmol/l and suburban 33.50 nmol/l. In rural, 27 nmol/l, urban, 33.4 nmol/l and 38.33 nmol/l vitamin D level observed in suburban sub clinical boys. Vitamin D level is significantly low in rural and urban sub clinical cases as compared to suburban (<0.05). In comparison with normal cases, rural and urban sub clinical cases had serum calcium and alkaline phosphatase level with significant differences (<0.05), but no significant differences were seen in suburban normal and abnormal cases (>0.05). Average phosphorus level in serum of all groups was in normal range with no significant difference (>0.05). On the other hand parathyroid hormone level was higher in sub clinical as compared to normal cases but non of all had level above normal range (>0.05) (Table-III). 

**Table-III:** Comparison of serum biochemical parameters in normal and sub clinical cases of different gender and groups

***Groups***	***Vitamin D*** *(nmol/l)* *Mean±SD*		***Calcium*** *(mg/dl)* *Mean±SD*		***Phosphorus*** *(mg/dl)* *Mean±SD*		***ALP*** *(U/l)* *Mean±SD*		***PTH*** *(pg/ml)* *Mean±SD*	
	***Boys***	***Girls***	***Boys***	***Girls***	***Boys***	***Girls***	***Boys***	***Girls***	***Boys***	***Girls***
Rural Normal	67.06 ±6.06	59.57 ±8.55	8.87 ±0.11	8.98 ±0.18	3.40 ±0.15	3.50 ±0.20	407.8 ±43.0	470 ±76.4	39.89 ±4.57	49.3 ±15.8
Rural Sub clinical	27 ±1.91	23.42 ±3.98	8.52 ±0.15	8.47 ±0.10	3.51 ±0.26	3.30 ±0.48	608 ±103	612.3 ±97.6	48.71 ±9.48	58.68 ±5.04
P value	0.0000	0.0000	0.0008	0.0000	0.33	0.13	0.0023	0.0001	0.054	0.050
Urban Normal	67.96 ±3.66	61.58 ±2.65	9.18 ±0.20	8.96 ±0.16	3.60 ±0.25	3.50 ±0.14	326.8 ±37.7	343.4 ±30.9	35.48 ±3.74	45.05 ±5.07
Urban Sub clinical	33.4 ±1.14	28.91 ±2.59	8.58 ±0.08	8.57 ±0.23	3.50 ±0.29	3.40 ±0.52	611 ±119	568 ±113	47.80 ±9.76	52.4 ±10.4
P value	0.0000	0.000	0.0000	0.0002	0.48	0.55	0.0062	0.0001	0.050	0.050
Suburban Normal	69.21 ±4.08	63.13 ±2.15	9.1 ±0.17	9.10 ± 0.13	3.65 ±0.24	3.40 ±0.44	338.1 ±34.8	397.5 ±44.6	29.76 ±5.07	39.17 ±3.89
Suburban Sub clinical	38.33 ±1.53	33.50 ±3.73	8.6 ±0.26	8.71 ±0.37	3.43 ±0.37	3.49 ±0.15	569 ±147	535 ±131	46.67 ±6.66	50.7 ±10.80
P value	0.0000	0.0000	0.064	0.058	0.42	0.64	0.11	0.052	0.051	0.051

ALP: Alkaline Phosphatase, PTH: Parathyroid hormone, Significant difference (<0.05), Non significant difference (>0.05)

## DISCUSSION

This study demonstrated that our geographical location is at great risk due to sub clinical rickets with prevalence rate of 27% in school students. Although this concern is present in student of all our territory but the major contribution is from students of rural schools 26(51%). Study conducted in Turkey found that urban populations are more effected by rickets^6^, the same observation was concluded by Zeghoud in his study.^7^

Alarming factors of low and poor sun rays availability was observed in rural students due to environmental uncertainty and traditional impacts. The present data highlighted that although this issue is present in students of both gender but girl’s contribution in this regard is maximum (71%). A study conducted in Tehran on girls show that, out of total 11% rickets cases low vitamin D level was seen only in 15(4%).^8^ Results from study which involved English population in England revealed that, 14% of total cases had low vitamin D level.^9^ Unfortunately Pakistani populations are not using foods fortification like many other countries.The socio economic status, such as family size and income of participant were almost similar.

Our investigation regarding vitamin D highlights that, all sub clinical cases had low vitamin D status. Daily proposed intake of vitamin D is 400 IU, calcium 1200 mg and phosphorus 1200 mg for adolescents group of age 11 to 18 years.^10^ In this study it was calculated that, all participants whether normal or with abnormal biochemical finding were taking less amounts of nutrients in their daily meals. Despite of that the low serum vitamin D level found in sub clinical rickets cases. The difference in serum vitamin D value might be the lack of synergistic effects due to low intakes and sunshine availability. The limited sunrays contributed to environmental uncertainty and traditional impacts due to unawareness of its benefits among populations.

The common source of vitamin D preparation in human being is sunlight.^11^ Asian foods contain high phytate content that affects vitamin D absorption.^12^ Other study related the low level of vitamin D with daily usage of bread which contain phytate^13^ and also reduce the absorption of calcium.^14^ Poor sun exposure is not responsible for low vitamin D.^6^ Our estimation recorded that, the low level of vitamin D present in 100%, calcium 55%, phosphorus 24% and high alkaline phosphatase 73% in sub clinical cases. Study on girls from Tehran shows that, 7% had elevated alkaline phosphatase level with normal or low calcium level but normal vitamin D value^15^ and similar results were seen in the studies of Africa and Bangladesh.^16^,^17^ High alkaline phosphates activity was found in Saudi children with low calcium level.^18^ A measurement of alkaline phosphatase is not an important parameter to investigate rickets^19^, also same conclusions were drawn by another study.^20^ Alkaline phosphatase level is the only way to evaluate rickets disease4 and it is also declared as the best indicator by other findings.^21^,^22^ Present study revealed that, the measurement of blood vitamin D level is the most reliable tool than alkaline phosphatase for the diagnosis of Subclinical rickets cases.

In this study interestingly none of the sub clinical cases had exceeded level of hormonal parathyroid from upper normal limits. This might be due to facts that phosphorus level either normal or low with no significant difference when compared to value of normal cases. Positive relation of vitamin D and parathyroid hormone is declared in study^23^ and same observation was found by others.^24^ In Finish study it was concluded that, vitamin D level of <40 nmol/l responsible for the elevation of parathyroid level.^25^ A study of clinical rickets highlighted that parathyroid hormone level is significantly increased from upper limits in vitamin D deficiency cases.^26^ Abnormal biochemical finding in sub clinical cases of this study might be due to lack of synergistic effect of sunshine and vitamin D.

## CONCLUSIONS

Sub clinical rickets is camouflagic rickets among hazarian school students of both genders especially in girls. We suggests that government should promote health education through experts on schools basis for the awareness and advantages of sunshine and nutritional intakes to overcome the problem of sub clinical rickets and their related problem in future. 

## References

[1] Brunvand L, Haga P, Tangsrud SE, Haug E (1995). Congestive heart failure cause by vitamin D deficiency. Acta Paediatr.

[2] Agarwal K (2005). Adolescent osteomalacia A case report of five years follows up. IJPMR.

[3] Holick MF (2003). Vitamin D: A millennium perspective. J Cell Biochem.

[4] Joiner TA, Foster C, Shope T (2000). The many faces of vitamin D deficiency rickets. Pediatr Rev.

[5] Methal A, Wahal DA, Benjour JP Vitamin D status in Asia. IOF committee of scientific Advisor (CSA), Nutritional Working group. Osteoporosis International.

[6] Uner A, Acar MN, Cesur Y, Dogan M, Caksen H, Temel H (2010). Rickets in healthy Adolescent in van, the eastern of Turkey. Eur J Gen Med.

[7] Zeghoud F, Delaveyne R, Rehel P, Chalas J, Garabedian M, Odievre M (1995). Vitamin D and pubertal maturation Interest and tolerance of vitamin D supplementation during the winter season. Arch Pediatr.

[8] Dahifar H, Faraji A, Yassobi S (2007). An asymptomatic rickets in girls adolescents. Indian J Pediatr.

[9] Margiloff L, Haris SS, Lee S, Lechan R, Dawson-Hughes B (2001). Vitamin D status of an outpatient clinic population. Calcif Tisseu Int.

[10] Herbert, Victor MD, Subak-Sharpe, Genell, J MD (1995). Adolescent total nutrition: The only Guide you’ll ever Need.

[11] Bhattacharya AK (1992). Nutritional rickets in the tropics. World Rev Nutr Diet.

[12] Robertson I, Kelman A (1977). Dunnigan MS Chapatti intake, vitamin D status and Asian rickets (Letter). Br Med J.

[13] Clements MR (1989). The problem of rickets in UK Asian. J Hum Nutr Dieter.

[14] Pettifor JM, Ross P, Wang J, Moodley G, Couper-Smith J (1978). Rickets in Children of Rural origion in South Africa Low dietry calcium factor. J Pediat.

[15] Dahifar H (2006). Impact of dietary and lifestyle on vitamin D in healthy student. J Med Invest.

[16] Thacher TD, Lghogboja SL, Sischer PR (1997). Rickets without vitamin D Deficiency in Nigerian children. Amb Child Health.

[17] Fischer PR, Rahan, Climma JP (1999). Rickets without vitamin D deficiency in Bangladesh. J Tropical Pediatr.

[18] Al-Jurrayan NA, El-Desouki ME, Al-HerbishAS, Al-MazyadAS, Al-Qhtani MM (2002). Nutritional rickets and osteomalacia in school children and adolescent. Saudi Med J.

[19] Pettifor J, Isdale JM, Sahakian J, Banson JDL (1980). Diagnosis of sub clinical rickets. Arch Dis Child.

[20] Goel KM, Sweet EM, Logan RW, Warren JM, Arneil GC, Shanks RA (1976). Florid and Sub clinical rickets among immigrant children in Glasgow. Lancet.

[21] Editorial (1971). Diagnosis of nutritional rickets. Lancet.

[22] Preece MA, Ford JA, McIntosh WB, Dunnigan M G, Tomlinson S, O'Riordan JIH (1973). Vitamin D deficiency in Asian immigrants to Britain. Lancet.

[23] Harkness L, Cromer B (2005). Low levels of 25-hydroxym vitamin D are associate with elevated parathyroid hormone in healthy adolescent females. Osteoporos Int.

[24] Park SY, Park SW, KangSK, Jun YH, Kim SK, Son BK (2007). Sub clinical rickets in breastfed infants. Korean J Pediatr.

[25] Outila TA, Kärkkäinen MUM, Lamberg-Allardt CJE (2001). Vitamin D status affect serum parathyroid hormone concentrations during winter in female Adolescents: associations with forearm bone mineral density. Am J Clin Nutr.

[26] Narchi H, Jamil M, Kulaylat N (2001). Symptomatic rickets in adolescence. Arch Dis Child.

